# Xuefu Zhuyu decoction for nonalcoholic fatty liver disease: A protocol for systematic review and meta-analysis

**DOI:** 10.1097/MD.0000000000025358

**Published:** 2021-05-14

**Authors:** Jiaxin Liu, Bo Dong, Lin Yang, Wei Huang, Songqi Tang

**Affiliations:** a College of Basic Medicine, Chengdu University of Traditional Chinese Medicine, Chengdu, Sichuan; b Hainan Medical University, Haikou, Hainan, China.

**Keywords:** meta-analysis, nonalcoholic fatty liver disease, protocol, systematic review, Xuefu Zhuyu decoction

## Abstract

**Background:**

Nonalcoholic fatty liver disease (NAFLD) includes nonalcoholic fatty liver (NAFL) and nonalcoholic steatohepatitis (NASH), which ranks only second to viral hepatitis and poses an increasingly serious challenge to global public health and economy. NAFLD has attracted more and more attention, but there is no drugs with exact curative effects are available. The commonly used drugs for the treatment of NAFLD in clinical practice are statins, such drugs, inevitably increase the burden on the live. Compared to statins, traditional Chinese medicines are believed to be “all natural” with fewer side effects, are associated with strong patient compliance. Accordingly, a great deal of clinical studies have shown that Xuefu Zhuyu decoction (XFZYD) can significantly improve the clinical symptoms and enhance the therapeutic effect. Meanwhile, a system review and meta-analysis are conducted by us to further clarify the effectiveness and safety of XFZYD for NAFLD.

**Methods:**

We will apply to database mainly range from the English literature searches Cochrane Library, PubMed, excerpt medica database, and Web of Science to the Chinese literature China national knowledge infrastructure, Chinese biomedical literature database, VIP, and Wanfang database, randomized controlled trials (RCTs) are enrolled to evaluate the effectiveness and safety of XFZYD in the treatment of NAFLD, the enrollment of RCTs is from the establishment of the database to February 01, 2021. Simultaneously we will retrieval clinical registration tests and grey literatures. The 2 researchers worked independently on literature selection, data extraction, and quality assessment. The dichotomous data is expressed in terms of relative risk, the continuous is represented by mean difference or standard mean difference, whether there is heterogeneity is the factor that determines the synthesis of data in fixed effect model or random effect model. Alanine aminotransferase (ALT) or Aspartic acid aminotransferase (AST) coupled with Glutamyltransferase (GGT) is considered as one of the main indicators of the NAFLD, while the pathology, imaging and diagnosis of metabolic syndrome are also auxiliary results. The last, meta-analysis was conducted by RevMan software version 5.3.

**Results:**

This study will provide evidence for treatment of NAFLD with XFZYD in terms of effectiveness and safety.

**Conclusion:**

This systematic review aims to confirm the efficacy and safety of XFZYD in the treatment of NAFLD.

**OSF registration number:**

DOI 10.17605/OSF.IO/7CWRK.

## 1 Introduction

Nonalcoholic fatty liver disease (NAFLD) is a common chronic liver disease associated with caloric excess and disordered metabolism, comprises a continuum of complicated pathological processes from simple steatosis to non-alcoholic steatohepatitis, cirrhosis and even hepatocellular carcinoma, which has seriously posed healthy burdens to the public.^[1]^

Hepatic steatosis is characterized by liver imaging or histology, and there is no other reason to explain it

1. history of drinking or equivalent alcohol intake for men <140 g/wk, women <70 g/wk;
2. except for viral hepatitis, drug-induced liver disease, autoimmune liver disease, total parenteral nutrition, hepatolenticular degeneration and other specific diseases that can cause fatty liver.

Another diagnostic criteria is unexplained serum ALT and (or) AST, GGT continue to increase for more than half a year, accompanied by metabolic syndrome related components, after losing weight and improving insulin resistance, fatty liver imaging and liver enzyme abnormalities improve or return to normal.^[2]^ NAFLD has been a leading cause of chronic metabolic disease, which is closed associated with obesity, diabetes and other metabolic diseases.^[3]^ The number of NAFLD patients worldwide is increasing year by year, among them, young people also account for a large proportion. Healthy diet, patient education and reasonable exercise are the main non-drug therapies to reduce NAFLD from the source.^[4]^

The western medicines (WMs) commonly used for the treatment of NAFLD include insulin sensitizer, anti-oxidants, protect liver medicine, lipid-lowering agent. The different kinds of drugs mentioned above are based on the various pathogenesis, such as lipid-lowering drugs are the main therapeutic drugs, among which statins as the representative drugs with antioxidant and anti-inflammatory effects.^[5]^ In view of the above-mentioned WMs interventions available but limited, at the same time, there is no magic bullet. Compared with WMs, traditional Chinese medicines have advantages of multiple targets, less adverse reactions, low price and so on. Xuefu Zhuyu decoction (XFZYD), which is well-known traditional Chinese formula, 11 compounds in XFZYD, namely Taoren, Honghua, Shengdi, Danggui, Niuxi, Chishao, Zhiqiao, Gancao, Chuanxiong, Jiegeng, and Chaihu, it has the effect of activating blood circulation and removing blood stasis, regulating qi and relieving pain. At present, some clinical studies have reported the effectiveness of XFZYD in the treatment of patients with NAFLD through effectively improving the blood lipid and liver function, as well as insulin resistance, antioxidant stress, cell protection.^[6–9]^ However, drugs both WMs and TCMs are mainly used in the liver for detoxification and metabolism, excessive or inappropriate drug intake will increase the burden of liver and hepatotoxicity. Therefore, drugs should be carefully selected for the treatment of NAFLD. We have found no systematic study on the efficacy and safety of XFZYD in the treatment of NAFLD, robust research is needed to address this efficacy and safety of XFZYD in the treatment of NAFLD. So in this study, we will systematically evaluate the efficacy and safety of XFZYD in treating DAFLD by a meta-analysis method, aiming to provide strong evidence-based medicine support for its further clinical applications.

## 2 Methods

### 2.1 Protocol and registration

The protocol has been registered on the Open Science Framework platform (https://osf.io/pcvuh/), registration number: DOI 10.17605/OSF.IO/7CWRK. This protocol was drafted and reported in accordance with the Preferred Reporting Items for Systematic Reviews and Meta-Analyses Protocols (PRISMA-P) guidelines.^[10]^ The final report will comply with the recommendations of the PRISMA Extension Statement for Reporting of Systematic Reviews Incorporating Meta-analyses of Healthcare Interventions.^[11]^

### 2.2 Ethics

We will not need individual data of each patient in the research as this is a systematic review. Therefore, institutional review board approval and ethics committee is not needed. Our purpose is to publish the results in a peer-reviewed journal. The final results of the review will provide information about the safety and efficacy of XFZYD and its modified forms in the treatment of NAFLD to help clinicians make decisions on clinical practice.

### 2.3 Inclusion criteria

#### 2.3.1 Type of study design

The study only chooses clinical randomized controlled trials of XFZYD for NAFLD published in both Chinese and English. However, animal experiments, reviews, case reports, and non-randomized controlled trials are excluded.

#### 2.3.2 Participants

The patients with NAFLD must meet the diagnostic criteria established by the recent advances in the diagnosis and treatment of nonalcoholic steatohepatitis^[12]^ and Interpretation of Chinese and foreign guidelines for the diagnosis and management of NAFLD.^[13]^ Exclude patients with alcoholic liver disease, liver diseases such as viral hepatitis, drug-induced hepatitis, autoimmune liver disease, or gastrointestinal malignancies. No gender, race, nationality and comorbidity are limited.

#### 2.3.3 Interventions

The control groups were treated with insulin resistance, oxidative stress and cell protection, the experimental groups were given different compatibility forms of XFZYD (exclude any other traditional Chinese medicine treatment) on the basis of the control groups. The dosage and treatment time of each group were not specified in this study. During the study, the 2 groups were not allowed to receive any other therapeutic intervention, which would literally affected the accuracy and scientific of outcome.

#### 2.3.4 Outcomes

The primary outcomes were conducted according to the NAFLD and recent guideline updates,^[14]^ Obvious effect: After 2 months of treatment, liver imaging or histology indicated that the patient's liver steatosis has greatly improved, ALT, AST, and GGT levels tended to be normal, there was no abnormality in blood lipid and liver function; Effective: After 2 months of treatment, patient's liver steatosis has improved to some extent, the levels of ALT, AST, and GGT slightly higher than normal, blood lipid and liver function have smaller difference with normal reference values; No effect: Two months after treatment, the patient's clinical indicators showed abnormal, what is more, liver imaging or histology results showed no remission of the disease or worse. Total effective rate = (obvious effect + effective rate)/total cases x100%.

### 2.4 Search methods

#### 2.4.1 Electronic searches

Literature retrieval is selected from the following databases: PubMed, MEDLINE, excerpt medica database, Cochrane Library, China National Knowledge Infrastructure, Wanfang data, Chinese Scientific Journals Database (VIP), and China biomedical literature database. We will choose studies published up to February 01, 2021, that is in line with the requirements of experiment. The search method used a combination of heading terms and free words, which should be as close to your research as possible. Search terms: Xuefu Zhuyu decoction, modified Xuefu Zhuyu decoction, Xuefu Zhuyu granules, Nonalcoholic fatty liver, Nonalcoholic fatty liver disease, etc. Taking PubMed as an example, the initial search strategy is shown in Table 1, which will be flexibly adjusted according to different databases.

**Table 1:** Search strategy of the PubMed.

Number	Search terms
#1	Nonalcoholic fatty liver disease [Mesh]
#2	Nonalcoholic fatty liver disease [Title/Abstract] OR Nonalcoholic fatty liver diseases [Title/Abstract] OR NAFLD [Title/Abstract] OR Nonalcoholic fatty liver [Title/Abstract]
#3	#1 OR #2
#4	Xuefu Zhuyu [Title/Abstract]
#5	Decoction [Title/Abstract]
#6	#4 AND #5
#7	randomized controlled trial [Publication Type]
#8	controlled clinical trial [Publication Type]
#9	Randomized [Title/Abstract]
#10	Randomly [Title/Abstract]
#11	#10 OR #11 OR #12 OR #13
#12	#3 AND #6 AND #11

#### 2.4.2 Searching other resources

At the same time, we will retrieve other resources to complete the deficiencies of the electronic databases, mainly searching for the clinical trial registries and grey literature about XFZYD for NAFLD on the corresponding website.

### 2.5 Data collection and analysis

#### 2.5.1 Selection of studies

**Figure 1:**
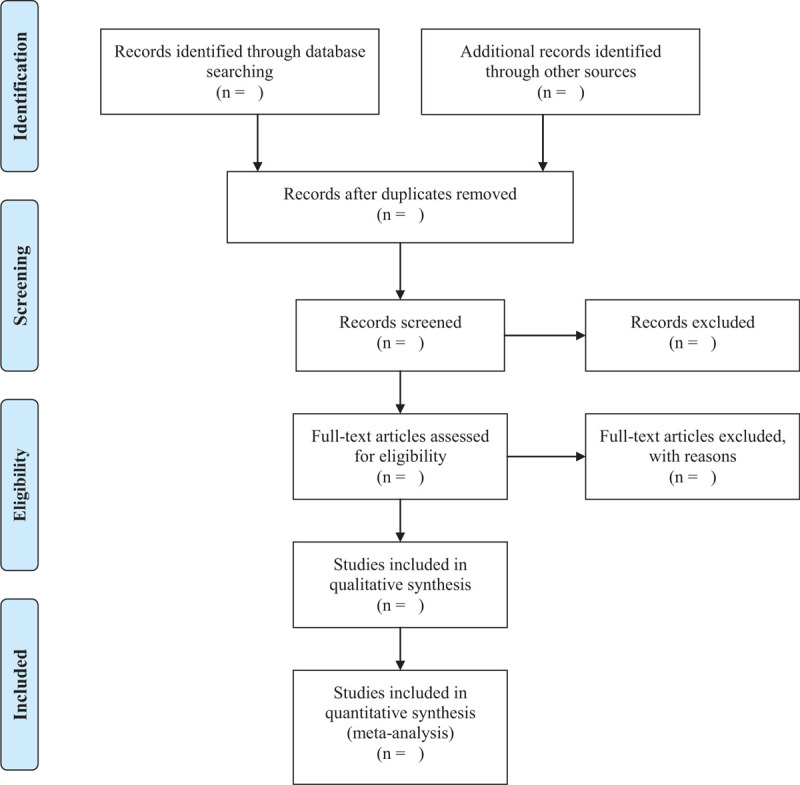
PRISMA flow diagram of the study selection process.

We used Endnote X8 software import all literatures that meet the requirements of the study. Firstly, 2 independent reviewers (Jiaxin Liu and Bo Dong) initially screened the literatures that did not meet the above-mentioned standards of the study by reading the title and abstract. Secondly, download the remaining literatures and read the full text carefully to further decide whether to include or not. Finally, the results were cross-checked repeatedly by reviewers. If there is a disagreement in the above process, we can reach an agreement by discussing between both reviewers or seek a third party's opinion (Lin Yang). Flow chart of the study selection (Fig. 1) will be used to show the screening process of the study.

#### 2.5.2 Data extraction and management

We build an excel form for data collection before data extraction. 2 reviewers (Jiaxin Liu and Lin Yang) filled in the data extraction which came from eligible studies. The main data extracted are as follows: title, author, year, fund source, sample size, age, sex, duration of disease, interventions, outcome measures, adverse reactions, etc. If there is any objection, the third researcher (Bo Dong) will be asked to assist in the judgment. Another common situation is something unclear in the study, you can contact the author of the communication directly for more detailed information. The above information was finally cross-checked by 2 reviewers.

#### 2.5.3 Assessment of risk of bias in included studies

The risk of bias is an assessment tool provided by the Cochrane Handbook which is used for quality assessment of RCTs. The following 7 items, including random sequence generation, allocation concealment, blinding of participants and personnel, blinding of outcome assessment, incomplete outcome data, selective outcome reporting, and other bias, above all are evaluated by 3 grades of “low bias,” “high bias,” and “unclear bias.” Which means each item is satisfactory, unsatisfied and vague respectively. The discrepancies will get a consistent conclusion by discussing between both reviewers or consult with the third-party.

#### 2.5.4 Measures of treatment effect

According to the purpose of our research, different evaluation methods correspond to the different efficacy indicators. For example, we will choose the effect scale indicator relative risk with 95% confidence interval (CI) to represent the dichotomous data. While the continuous data is expressed as mean difference or standardized mean difference with 95% CI depending on whether the measurement scale is consistent or not.

#### 2.5.5 Dealing with missing data

In the process of the statistical data, the reviewers accidentally found that data in some documents was missing, the top priority is to contact the first author or correspondent author via email or telephone to obtain related information. If not, the initial analysis will be conduct to synthesize the available data. Fortunately, we can use sensitivity data analysis to determine the extent of the impact of missing data on the entire study results.

#### 2.5.6 Assessment of heterogeneity and data analysis

The Chi-Squared test and *I*^2^ test were used to detect the heterogeneity of the included studies. When *P* < .1, it indicates that there is heterogeneity between the studies. The larger the *I*^2^, the greater the heterogeneity, *I*^2^ > 50%, it means that there is a large heterogeneity. The combined analysis of research results without statistical heterogeneity (*I*^2^ < 50%, *P* > .1) adopts a fixed-effect model. When there is statistical heterogeneity (*I*^2^ ≥ 50%, *P* < .1), a random effect model is used to analyze. We used Review Manager software version 5.3, which is created by the Cochrane Collaboration for data synthesis and analysis. If a meta-analysis cannot be performed, it will be replaced by a general descriptive analysis.

#### 2.5.7 Subgroup analysis and sensitivity analysis

If the results of the study are heterogeneous, the possible causes of the heterogeneity are analyzed, and sub-component analysis is used. If the heterogeneity cannot be eliminated, the data is from those who can be combined from a clinical perspective, the random effects model is used for combined analysis, and the results are interpreted carefully. We will conduct a subgroup analysis for different reasons. Heterogeneity is manifested in the following several aspects, such as race, age, sex, different intervention forms, pharmaceutical dosage form, dosage, treatment course. When there are low-quality studies in the subgroup, low-quality studies are excluded for sensitivity analysis, and then merge the data to assess the impact of sample size, study quality, statistical method, and missing data on results of meta-analysis.

#### 2.5.8 Reporting bias

If there are more than10 studies in the meta-analysis, the funnel chart will be evaluated, and the distribution of the collected clinical research data will be observed to determine whether there is a result of publication bias.

#### 2.5.9 Grading the quality of evidence

We using the Grading of Recommendations Assessment, Development and Evaluation (GRADE) system to evaluate the quality of evidence for the entire study, which is established by the World Health Organization and international organizations.^[15]^ Giving the risk of bias, imprecision, inconsistency, indirection, and publication bias, the GRADE system divides the quality of evidence into 4 levels: high, medium, low, and very low. The GRADE profiler 3.2 will be employed for analysis.

## 3 Discussions

Nonalcoholic fatty liver disease (NAFLD) is becoming the most common chronic liver disease, it is defined as at least 5% steatosis observed in the hepatocytes on either histology or by imaging methods. NAFLD is a complex and multisystemic disease, and its pathogenesis involves many factors, including genetic factors, environmental factors, and metabolic factors.^[16]^ Effective medications for NAFLD, which are more direct and effective, are priorities because of the low feasibility of lifestyle modification. Ideally, the drugs should not damage extra hepatic organs, should have low side effects, and should benefit NAFLD. No effective interventions are currently available. Herein, most promising drugs of WMs in or beyond different phase clinical trials.

There are many reports on the treatment of NAFLD with traditional Chinese medicine, XFZYD as one of the famous Chinese medicine prescriptions, is founded by Qingren Wang in the Qing Dynasty. Literature reported XFZYD can improve liver inflammation in terms of pathology, as well as reduce liver steatosis, anti-apoptosis of hepatocytes.^[17,18]^ The most important is that XFZYD has the advantage of low price and long-lasting effect. However, there has been no systematic review or meta-analysis to evaluate the therapeutic effect. Therefore, we conduct this systematic review to further evaluate the effectiveness and safety of XFZYD for NAFLD, aiming to provide more ample scientific basis in the treatment of NAFLD for clinicians clinical practice.

RCTs are regarded as the gold standard for clinical evaluation of drugs and are at the top of the evidence-based medicine evidence evaluation pyramid. The large-sample, multi-center, random, double-blind, high-quality RCTs are to evaluate the effectiveness and safety of TCMs in the treatment of NAFLD. It is recommended that it be actively carried out in the future. At the same time, consideration should be given to improving the design and implementation of Chinese medicine RCTs to obtain high-level evidence for the treatment of NAFLD with XFZYD.

## Author contributions

**Formal analysis:** Lin Yang.

**Investigation:** Bo Dong.

**Resources:** Bo Dong.

**Supervision:** Wei Huang.

**Writing – original draft:** Jiaxin Liu.
